# Genetically regulated hepatic transcripts and pathways orchestrate haematological, biochemical and body composition traits

**DOI:** 10.1038/srep39614

**Published:** 2016-12-21

**Authors:** Siriluck Ponsuksili, Nares Trakooljul, Frieder Hadlich, Fiete Haack, Eduard Murani, Klaus Wimmers

**Affiliations:** 1 Research Unit ‘Functional Genome Analysis’, Leibniz Institute for Farm Animal Biology (FBN), Wilhelm-Stahl-Allee 2, D-18196 Dummerstorf, Germany; 2 Research Unit ‘Genomics’, Leibniz Institute for Farm Animal Biology (FBN), Wilhelm-Stahl-Allee 2, D-18196 Dummerstorf, Germany

## Abstract

The liver is the central metabolic organ and exhibits fundamental functions in haematological traits. Hepatic expression, haematological, plasma biochemical, and body composition traits were assessed in a porcine model (n = 297) to establish tissue-specific genetic variations that influence the function of immune-metabolism-correlated expression networks. At FDR (false discovery rate) <1%, more than 3,600 transcripts were jointly correlated (r = |0.22–0.48|) with the traits. Functional enrichment analysis demonstrated common links of metabolic and immune traits. To understand how immune and metabolic traits are affected via genetic regulation of gene expression, eQTLs were assessed. 20517 significant (FDR < 5%) eQTLs for 1401 transcripts were identified, among which 443 transcripts were associated with at least one of the examined traits and had cis-eQTL (such as ACO1 (6.52 × 10^−7^) and SOD1 (6.41 × 10^−30^). The present study establishes a comprehensive view of hepatic gene activity which links together metabolic and immune traits in a porcine model for medical research.

The liver is of particular interest given its vital roles in maintaining homeostasis and health and regulating nutrient utilization. Accordingly, insights into the regulation of liver expression profiles potentially have implications for traits associated with physical and metabolic integrity. Traits related to fatness and body composition are important not only as economic factors in pork production but also because of their association with serious diseases in humans^1^,^2^. Pigs share many similarities with humans in terms of their physiology and genome and therefore provide a good model for medical research including studies on the transplantation of organs^3^.

Accordingly, we used expression analyses in the liver to identify genes associated with plasma haematological and biochemical traits that might serve as biomarkers of “liver functioning” and as surrogate traits for immune and metabolic status. However, variations in complex traits depend not only on the expression profile of single hepatic genes but also on networks of genes and transcripts. Therefore, we further performed a weighted gene co-expression network analysis (WGCNA) to systematically assess the pathways in which co-expressed genes are interconnected. Thus, we obtained a more precise set of parameters that might serve as either key biomarkers of acute metabolic and immune status mediated through hepatic functions, or as predictors of genetically determined metabolic and immune capabilities.

Expression-QTL (eQTL) analysis integrates gene expression levels and genome-wide genotyping information to identify genetic variations associated with changes in gene expression. Variations in complex traits largely reflect polymorphisms affecting regulatory sequences, rather than coding sequences. Knowledge of the position of the analyzed genes and markers enables the differentiation of cis- and trans-eQTLs^4^. We have previously shown that the detection of trait-dependent expressed genes in a relevant tissue facilitates the identification of genes associated with complex traits, such as muscle and meat properties and coping behaviors; these genes represent strong candidate genes^5^,^6^. Studies of eQTLs in the human liver have led to the identification of genes associated with clinical phenotypes and provide a foundation for pharmacogenomics^7^.

Given the central role of the liver in controlling the homeostasis of the metabolic and immune systems, body composition and all fat traits, we hypothesized that the variation of these traits might largely reflect genes and metabolic pathways active in the liver. Herein, we characterized hepatic transcription profiles and the genetic regulation of their expression in association with immune and metabolic traits in pigs. The analyses of trait-correlated hepatic expression, co-expression interactions and eQTL-detection provide a common link for haematological, biochemical, and clinical-chemical biomarkers of hepatic functions contributing to metabolic homeostasis, innate defense and resilience. This knowledge provides a rational basis not only for understanding pig physiology but also for pig models used in human medical research.

## Results

### Hepatic trait-correlated expression

The expression levels of 24904 liver transcripts in 297 individuals were correlated with biomarkers of immune and metabolic status, including haematological, biochemical and body composition traits. All measurements and descriptions of these traits are shown in Supplementary Table 1. Haematological traits refer to three components: leukocytes (white blood cell count: WBC, lymphocyte count: LYM), erythrocytes (red blood cell count: RBC, hemoglobin concentration: HGB, hematocrit level: HCT, mean corpuscular volume: MCV, mean corpuscular volume: MCH, mean corpuscular hemoglobin concentration: MCHC, red distribution width: RDW), and platelets (platelets: PLT, mean platelet volume: MPV, plateletcrit: PCT), and the respective cell counts are markers of immune and/or inflammatory responses^8^,^9^. At a significance threshold of an FDR < 1%, we detected 5387 transcripts showing correlations with at least one of the 12 examined haematological traits. The correlation between expression levels and haematological traits ranged from |0.22–0.48|. Most transcripts were correlated with leukocytes and erythrocytes, and a few transcripts were associated with platelets.

**Figure 1:**
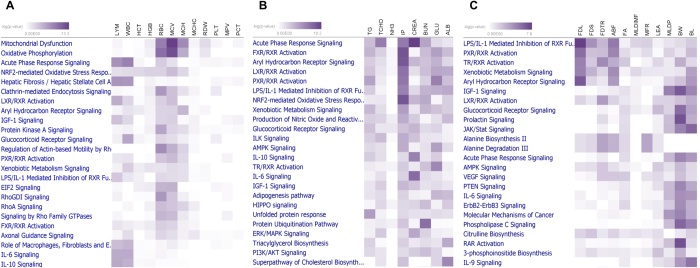
Canonical pathways of transcripts correlated with (**A**) haematological traits, (**B**) biochemical traits, and (**C**) body composition phenotype. Heatmap displaying the correlation of transcripts related to canonical pathways with phenotypes; intensity of color indicates significance from light to dark.

To further refine the functional annotation of the sets of genes showing trait-correlated expression, their assignment to canonical pathways was explored using the Ingenuity Pathway Knowledge Base. Biological functions associated with trait-correlated expression are displayed in Fig. 1A and Supplementary Table 2A. Acute phase response signaling and hepatic fibrosis/hepatic stellate cell activation were correlated with LYM and WBC. Most of the erythrocyte-related traits were correlated with oxidative phosphorylation, mitochondrial dysfunction and Ephrin A signaling. The coagulation system and complement system were correlated with platelet-related traits.

The phenotypes of 8 biochemical traits (albumin: ALB, ammonia nitrogen: NH3, blood urea nitrogen: BUN, total cholesterol: TCHO, triglyceride: TG, glucose: GLU, inorganic phosphorus: IP, creatinine: CREA) were used to examine correlations with the expression profiles of the liver. At a significance level of an FDR < 1%, 6321 transcripts were correlated with a minimum of one biochemical trait at r = |0.22–0.41|. Canonical pathways such as acute phase response signaling showed the closest association with CREA, TCHO and BUN. TCHO-, TG- and GLU-correlated genes were associated with common canonical pathways of PXR/RXR activation, FXR/RXR activation and TR/RXR activation (Fig. 1B; Supplementary Table 2B).

Body composition traits were examined to characterize metabolic end product phenotypes. In total, 11 traits, including 6 fat traits (fat depth at shoulder: FDS, fat depth at tenth rib: FDTR, loin fat depth at loin: FDL, average back fat: ABF, fat area: FA, Intramuscular fat content: MLDIMF), 3 muscle traits (loin eye area: LEA, protein content: MLDP, muscle to fat ratio: MFR), body weight (BW) and body length (BL), were used. At a significance level of an FDR < 1%, 2571 transcripts were correlated with at least one body composition trait at r = |0.22–0.44|. The list of transcripts correlated with fat traits was associated with the canonical pathways of LPS/IL-1-mediated inhibition of RXR function, TR/RXR activation, LXR/RXR activation, PXR/RXR activation and xenobiotic metabolism signaling. For the muscle traits, the list of correlated transcripts was enriched for IGF-1 signaling, alanine biosynthesis II, alanine degradation III, phenylalanine degradation I and glucocorticoid receptor signaling (Fig. 1C; Supplementary Table 2C).

**Figure 2:**
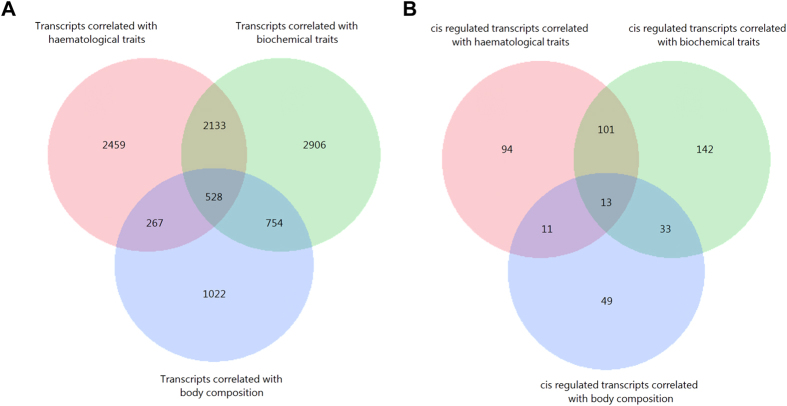
Venn diagrams displaying numbers of trait-correlated transcripts. (**A**) Number of liver transcripts correlated with haematological and biochemical traits as well as body composition traits. (**B**) Number of liver transcripts correlated with haematological and biochemical traits as well as body composition traits with cis eQTL effect.

Altogether, 10064 out of 24904 transcripts were correlated with at least one of the surrogate traits of immune and metabolic status, with considerable overlap among the groups of traits (Fig. 2A). In fact, 2661 transcripts were common between haematological and biochemical traits, and 1282 transcripts were common to biochemical and body composition phenotype traits, while 795 transcripts were shared between haematological and body composition phenotype traits, and 528 transcripts were common to all immune and metabolic status traits. The numbers of transcripts that were only associated with haematological, biochemical and body composition traits were 2459, 2906 and 1022, respectively.

### Hepatic co-expression and trait-associated co-expression modules

**Figure 3:**
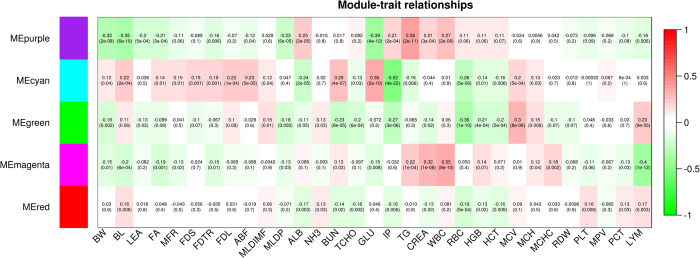
Correlation matrix of module eigengene values obtained for mRNAs and phenotypes. Weighted gene co-expression network analysis (WGCNA) groups genes into modules based on patterns of gene co-expression. Each of the modules was labelled with a unique color as an identifier. 5 modules show highly significant correlation with body composition, biochemical and haematological traits. Within each cell, upper values are correlation coefficients between module eigengenes and the traits; lower values are the corresponding p-values.

**Figure 4:**
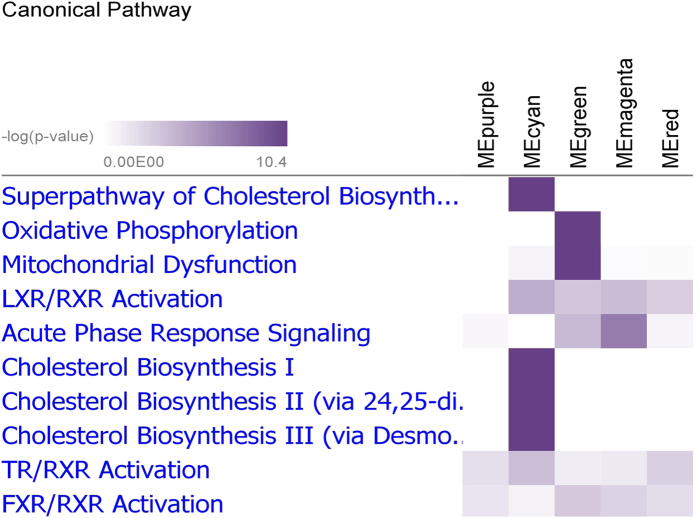
Canonical pathways of the modules whose eigenvalues correlated with the surrogate traits. Heatmap displaying the correlation of modules of trait-correlated and co-expressed transcripts with canonical pathways; intensity of color indicates significance from light to dark.

We explored transcriptional changes not only at the level of individual genes but also in terms of gene interactions. Thus, a WGCNA was performed using the transcriptome data from 24904 liver transcripts. Five modules were highly correlated with phenotypes, as shown in Fig. 3. The co-expressed transcripts in each module were assigned to canonical pathways. The magenta module was highly correlated with leucocyte counts, whereas the green module was highly correlated with erythrocyte counts. The top highly connected hub genes in the magenta module were *SOCS3, LOC100154449, JUNB, BTG2* and *IL4R*. The transcripts in the magenta module were associated with acute phase response signaling, whereas the transcripts in the green module were core components of the mitochondrial oxidative phosphorylation complexes encoded by the mitochondrial genome (Fig. 4). The highly connected hub genes in the green module were *MT-ATP6, MT-ND4L, MT-CO3, MT-ATP8* and *MT-ND5*. Platelet counts were modestly correlated with the red module, which was enriched for genes belonging to HIPPO signaling. The highly connected hub genes in the red module were *LOC102159151, LOC102159016, CD86, LOC100511343* and *MAB21L3*. Body composition traits associated with muscle mass were correlated with the purple module, whereas fat traits were more correlated with the cyan module. The 85 transcripts in the purple module were enriched in PXR/RXR activation, in contrast with the 65 transcripts in the cyan module, which encoded many genes associated with cholesterol biosynthesis (Fig. 4). The most connected hub genes in the cyan module were *ACACA, ACSS2, EBP, GPAM, GPAT* and *THRSP*. Biochemical traits such as ALB, GLU and TG were correlated with the purple module. *PPP1R3C, G6PC, PPP1R3B, SLC25A25, SGK1* were the most connected genes in the purple module.

### Hepatic eQTLs

A numerical summary of the whole-genome association study of gene expression levels in the liver (eQTL) is shown in Table 1. In total, 20517 significant eQTLs, corresponding to 1401 probe sets, reached the threshold of an FDR < 5% (*p* < 10^−7^). At this significance level, 11366 SNPs were associated with the expression of 1075 annotated transcripts, and 6865 eQTLs were identified as cis, belonging to 1028 probe sets (814 annotated transcripts) (Supplementary Table 3).

### Hepatic cis-eQTL and plasma haematological traits

**Figure 5:**
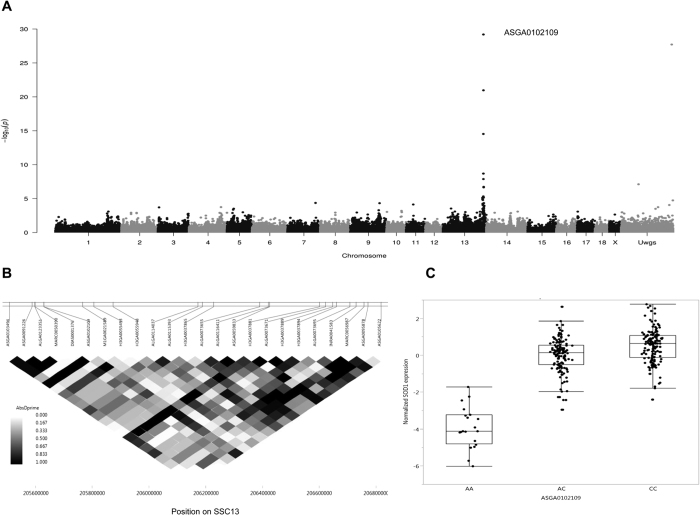
Genome wide association of *SOD1* transcript level. (**A**) Manhattans plot of cis-eQTL of *SOD1* and highly associated with SNP ASGA0102109 at 205.6 Mb of pig chromosome 13 (SSC13). (**B**) Linkage disequilibrium regions range from 205–206 Mb of SSC13 while *SOD1* star position at 205.6 Mb. (**C**) Genotype of ASGA0102109 loci show associated with *SOD1* transcript levels.

**Figure 6:**
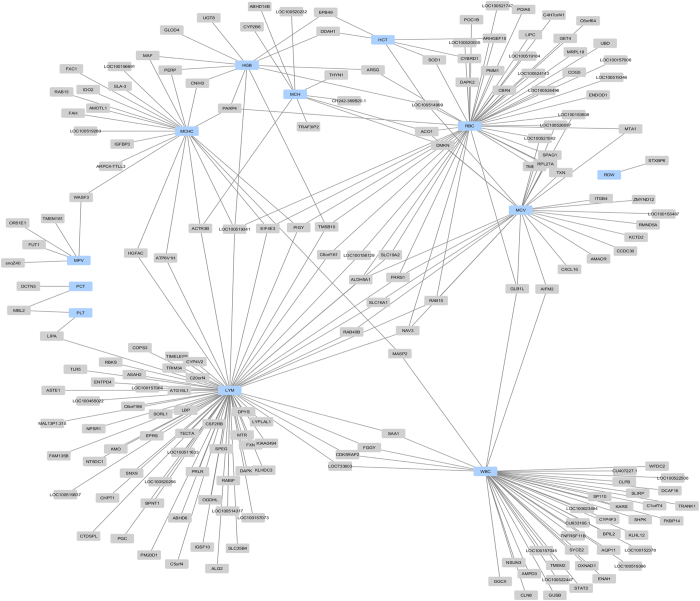
A network of all liver hepatic transcripts with cis-eQTLs and expression levels correlated with haematological traits. Each node represents a haematological traits (blue) connected with various transcripts (grey). Some of the transcripts are connected with more than one node.

The expression levels of 341 transcripts were significantly correlated with haematological traits associated with SNPs, revealing 2,439 eQTLs. Focusing on cis regulation, 808 cis-eQTLs were identified from 219 transcripts that were significantly correlated (FDR < 1%) with one of haematological traits (Supplementary Table 4). For traits associated with red blood cells we identified 47 annotated transcripts with eQTLs showing trait-correlated expression with RBCs, including 6 for HCT, 12 for HGB, 27 for MCV, 9 for MCH, 22 for MCHC and 1 for RDW loci. Superoxide dismutase 1 (*SOD1*) was one of the transcripts that were highly negatively correlated with HCT and RBC and exhibited a cis-eQTL with a *p*-value ranging from 6.08 × 10^−10^ to 6.41 × 10^−30^ (Fig. 5A). SNP ASGA012109 located in linkage disequilibrium regions of 205–206 Mb of chromosome 13 (Fig. 5B) was highly associated with *SOD1* levels (Fig. 5C). Most of the annotated transcripts (72 transcripts) were correlated with the LYM and WBC. In total, we identified 5 annotated transcripts correlated with MPV and 2 annotated transcripts correlated with PLT and PCT. All liver transcripts with cis-eQTLs whose expression levels were correlated with haematological traits are presented as a network in Fig. 6.

### Hepatic cis-eQTL and plasma biochemical traits

**Figure 7:**
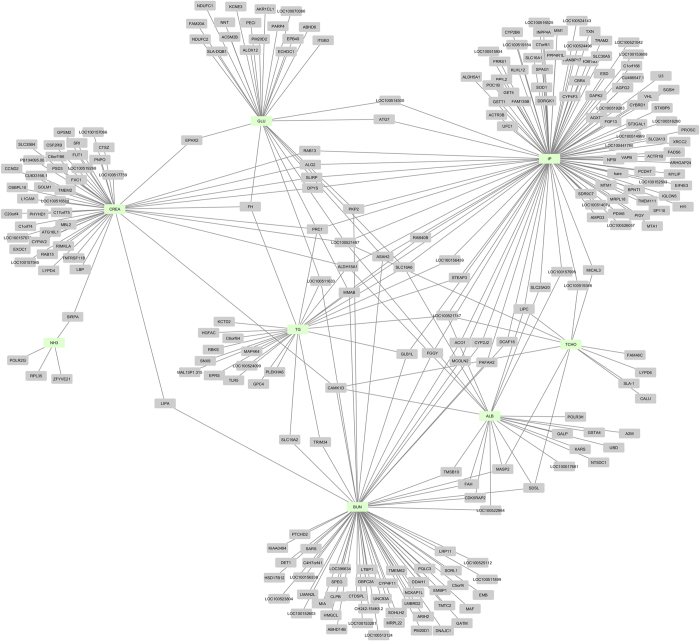
A network of all liver hepatic transcripts with cis-eQTLs and expression levels correlated with biochemical traits. Each node represents a biochemical traits (green) connected with various transcripts.

There were 447 transcripts correlated with biochemical traits at a significant level of a 1% FDR, which were also associated with 3554 eQTLs, including 1148 cis-eQTL of 289 transcripts (Supplementary Table 5). Most of the transcripts were correlated with IP (120) followed by BUN (73) and CREA (62). We identified 24 transcripts correlated with ALB, 29 transcripts correlated with GLU, 33 transcripts correlated with TG, 12 transcripts correlated with TCHO and only 4 transcripts correlated with NH3. Some transcripts were correlated with more than one biochemical trait. A corresponding network of transcripts is shown in Fig. 7.

### Hepatic cis-eQTLs and body composition

**Figure 8:**
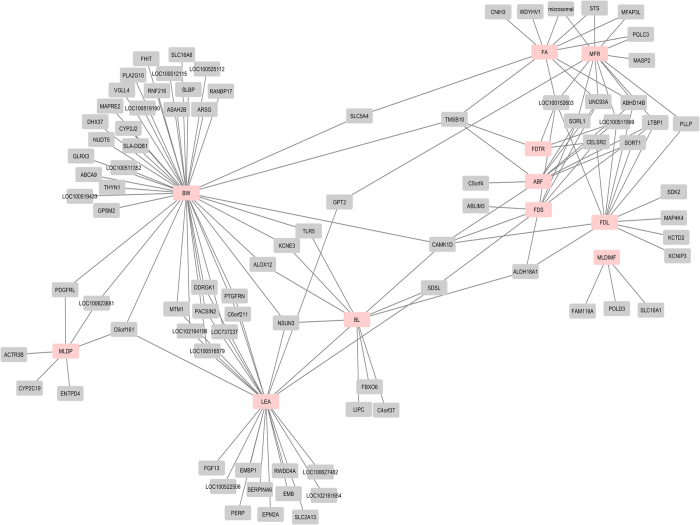
A network of all liver hepatic transcripts with cis-eQTLs and expression levels correlated with body composition traits. Each node represents a body composition traits (red) connected with various transcripts (grey). Some of the transcripts are connected with more than one node.

A total of 152 transcripts whose expression levels were correlated with body composition traits presented 1582 eQTLs, including 645 cis-eQTLs of 106 transcripts (Supplementary Table 6). Most of the transcripts were correlated with carcass traits (body weight (172) and length (80)) at slaughter. The number of transcripts correlated with fat/muscle traits ranged from 6–104. A network of these transcripts is shown in Fig. 8.

### Genetic regulation of liver transcripts contributes to common immune and metabolic complex traits

The 1028 transcripts with cis-eQTL primarily belonged to the canonical pathways of LPS/IL-1 mediated inhibition of RXR function, xenobiotic metabolism signaling and glutathione-mediated detoxification. A total of 443 of the 1028 transcripts were correlated with one of these surrogate traits of immune and metabolic status with considerable overlap among the group of traits (Fig. 2B). A total of 114 transcripts overlapped between haematological and biochemical traits, while 46 transcripts were shared between biochemical traits and the body composition phenotype, and only 24 transcripts overlapped between haematological traits and the body composition phenotype. Among the 443 transcripts, 13 were associated with the all types of traits belonging to 10 annotated genes, including *TLR5, KCTD2, SLC16A1, ACTR3B, SORL1, C5orf4, ABHD14B, TMSB10, MASP2* and *LIPC*.

## Discussion

The results of the present study provide a comprehensive dissection of the liver transcriptional landscape corresponding to clinical immune and metabolic markers. Furthermore, this is the first report of markers of immune and metabolic status and body composition correlated with hepatic mRNA expression profiles. Additionally, the co-expression of whole transcripts was analyzed using well-designed and characterized Snowball arrays^10^. WGCNA groups genes with similar profiles into modules and hub nodes that play important roles in many networks, and highly connected hub genes play an important role in biology and regulation as well^11^,^12^. Many hub genes identified in the present study, such as *SOCS3* and *JUNB* in the magenta module, have been reported as a key transcriptional modulators of macrophage phagocytosis and activation^13^. Hub genes in the purple module such as *PPP1R3B* and *PPP1R3C* act as glycogen phosphorylases in liver glycogen metabolism^14^. Other hub genes in the cyan module, such as *ACACA, GPAM, EPB* and *ACSS2*, play a significant role in lipid metabolism.

We assessed whole-genome expression in the liver to establish tissue-specific genetic variations that influence the function of immune-metabolism-correlated genes. The liver is a heterogeneous tissue with different cell types; more than 80% are hepatocytes, and the minor populations are cholangiocytes, stellate cells, endothelial cells and several hematopoietic cells. Variation in transcript abundance is possibly due to either variation of cell population or the change of cell functions. However, the tissue sampling was done from an identical anatomic site of healthy mature livers with a normal cell composition. Moreover, hepatic cell composition is stable and this study addresses the link of genetic variation and transcript abundance, with the first being independent from cell type. The variations represent the causal link between SNPs, gene expression and immune-metabolic status. To determine the genetic impact on expression, we focused on cis-eQTLs by considering SNPs located in genomic windows near the transcripts. These regions include promoters, enhancers and UTRs encompassing transcription factor-binding sites and regulatory elements^15^,^16^.

### Genetic regulation of liver transcripts and immune status

#### Erythrocytes

A potential role of erythrocytes as surrogate biomarkers of hepatic mitochondrial oxidative status in diverse oxidative conditions has been reported in an animal model^17^. Previous studies have reported that defects in enzymes involved in hepatic heme biosynthesis play a significant role in mitochondrial energetic metabolism, as shown in the Hmbs (−/−) mouse model, where mitochondrial respiratory chain complexes I, II and, III are significantly reduced^18^. Here, we demonstrated that hepatic energetic metabolism was correlated with haematological traits such as MCV, MCH and RBC.

In addition, the majority of transcripts correlated with erythrocyte traits showing significant cis-eQTL, such as Aconitase 1 (*ACO1*), Superoxide dismutase 1 (*SOD1*), and solute carrier family 19 member 2 (*SLC19A2*) were also related to the oxidative stress. ACO1 is a bifunctional, cytosolic protein involved in the control of iron homeostasis and the TCA cycle for energy metabolism^19^. In the present study, the transcript levels of *ACO1* were correlated with erythrocytes traits (RBC, MCH and MCV) with highly significant cis-eQTLs. Notably, iron homeostasis plays an essential role in the oxidative status of the cell, as pathological accumulation of iron might lead to the generation of additional reactive oxygen species (ROS) and oxidative stress^20^,^21^. SOD1 is one of the most important antioxidants and has a crucial impact on the lifespan and quantity of red blood cells in peripheral blood, as shown in *Sod1*-deficient (knockout) mice^22^. Accordingly *Sod1* knockout mice are unable to compensate for oxidative stress triggers, resulting in dysfunction of the proteasomal system and accelerated accumulation of damaged proteins. Mutation of the mouse *Sod1* gene (knockout) decreases the number of erythrocytes and quantity of peripheral blood red blood cells^22^. A recent study reported that oxidative-stress triggers dysfunction of the proteasomal system and accelerates the accumulation of damaged proteins, leading to a shortened lifespan of RBCs and, hence, anemia in Sod1-deficient mice^23^. In the present study *SOD1* was found to be highly negatively correlated with HCT and RBC and exhibited a cis-eQTL. The associated SNPs were located between 727 bp to -496 bp from the start codon of *SOD1*. SLC19A2 encodes a thiamine transporter protein. Thiamine also plays a role in reducing cellular oxidative stress via bridging the energy-producing glycolytic and pentose phosphate metabolic pathways, which is critical for generating ferric-reducing/antioxidant chemical-reducing power in cells^24^,^25^. Mutation of the mouse *Slc19a2* gene (homozygous knockout) also decreases the number of erythrocytes in mice^26^. In the present study, the cis-eQTL of *SLC19A2* was positively correlated with RBC and negatively correlated with LYM, consistent with a previous report. Apparently, the role of the liver in controlling the oxidative status of mitochondria (i.e. balancing metabolism induced production of ROS and the immediate detoxification of reactive intermediates) has a crucial effect on erythrocyte traits.

We identified significant associations of genetic variants with ribosomal protein gene and erythrocyte traits. *RPL27A* have a strong cis-eQTL (1.09 × 10^−25^–1.41 × 10^−11^) and its expression levels were found to be correlated with erythrocyte traits, such as RBC and MCV. A group of pathologies have been associated with ribosome defects, among which Diamond-Blackfan anemia (DBA) is the best studied, showing common clinical features including anemia, low reticulocyte counts, and macrocytic erythrocytes^27^,^28^,^29^. Hematocrit level (HCT) and RBC were shown to be highly correlated with Rho guanine nucleotide exchange factor 10 (*ARHGEF10*), and SNPs located within the 1 Mb windows of this gene were associated with its expression levels in the present study. The *ARHGEF10* gene was significantly associated with atherothrombotic stroke, which occurs when a blood clot forms on an atherosclerotic plaque within a blood vessel and blocks blood flow^30^. The SNP rs4376531 affects *ARHGEF10* transcriptional activity, reflecting differences in Sp1-binding affinity^30^. We demonstrated a relationship between erythrocytes and the genetically regulated expression levels of *ARHGEF10,* but the function remains elusive.

#### Leukocytes

A major function of the liver is to manufacture and secrete substances that are transported via the systemic circulation. Indeed, local inflammatory or injurious processes in the liver, e.g., after partial hepatectomy and during hepatic fibrosis, largely affect leukocyte-mediated activity throughout the body^31^. Kupffer cells, which are specialized macrophages in liver, are activated upon liver infection and injury, and leukocytes, which release pro-inflammatory cytokines such as tumor necrosis factor (TNF), and reactive oxygen^32^, also regenerate IL-6 and IL-10.

We identified *SAA1*, which encodes serum amyloid A (SAA), a major acute-phase protein primarily synthesized and secreted in the liver, as a strong cis-eQTL and demonstrated that the expression of *SAA1* was correlated with LYM. SAA has been reported as a chemoattractant for phagocytes and mast cells^33^,^34^, exhibits cytokine-like properties^35^ and induces T lymphocyte migration and adhesion to endothelial cells^36^ during the acute-phase response. LBP encodes a protein involved in the acute-phase immunologic response to Gram-negative bacterial infections and is primarily induced in the liver^37^. A previous study indicated that mutations in the lipopolysaccharide-binding proteins impair innate immunity through reduced binding to LPS and lipopeptides and reduced cytokine responses and concentrations in pneumonia^38^. Here, we identified SNPs surrounding the LBP transcript that were associated with its expression levels and LBP expression levels were correlated with the LYM. Thus, not only structural variation but also the expression of LBP might play a significant role in innate immune response. SORL1, also known as LR11, encodes a mosaic protein belonging to the vacuolar protein sorting 10 (VPS10) domain-containing receptor family and the low-density lipoprotein receptor (LDLR) family. Soluble LR11 (sLR11) is released through proteolytic shedding. Circulating sLR11 is a biomarker for atherosclerosis, coronary stenosis, diabetic retinopathy and acute leukemia^39^,^40^,^41^,^42^. A role for CD9 in the shedding of LR11 in leukocytes has also been reported^43^. We identified a cis-eQTL for *SORL1* and showed negative correlation of its expression with LYM. *TNFRSF11B* (tumor necrosis factor receptor superfamily member 11b) encodes a protein belonging to the TNF-receptor superfamily. Studies in a mouse counterpart also suggested that this protein and its ligand play a role in lymph node organogenesis and vascular calcification^44^,^45^. A cis-eQTL of *TNFRSF11B* was also identified in the present study, and its transcription levels were highly correlated with WBC (r = 0.329, p = 1.85 × 10^−8^).

#### Platelets

Platelets are small anucleate blood elements involved in hemostasis and thrombosis and immune responses^46^. The intravenous injection of low levels of lipopolysaccharide (LPS) into mice induces the accumulation of platelets in the liver, which eventually return to the circulation or undergo degradation^47^. The complement system is involved in this degradation. The interactions between platelets and the complement system are complex; platelets can activate complement and vice-versa^48^. In the present study, only a few liver transcripts were correlated with platelets, and most of these transcripts belonged to the coagulation system and the complement system. Mannose-binding lectin (*MBL2*) encodes mannose-binding protein and is capable of activating the complement pathway. The expression of complement factors during infection is tissue specific, and complement expression in the liver results from a systemic acute-phase response to infection^49^. Polymorphisms in *MBL2* have been associated with susceptibility to infectious diseases and immune traits as well as complement activity^50^,^51^,^52^. In the present study, a significant positive correlation between platelets and *MBL2* transcript levels (PCT and PLT, r = 0.25 *p* = 4.19 × 10^−5^) was detected. Although some reports have emphasized a role of platelet interactions with the complement system, the direct role of *MBL2* remains unknown^53^,^54^. The genetic regulation of *MBL2* transcripts might affect platelet traits and, ultimately, immune status. The α1,2-fucosyltransferase I (FUT1) enzyme is important for the biosynthesis of membrane glycoproteins that play a role in various inflammatory reactions^55^. In pigs, *FUT1* is associated with resistance to E.*coli* F18 infection and the expression of various defense pathways^56^,^57^. In the present study, *FUT1* was shown to exhibit a cis-eQTL (*p* = 2.2 × 10^−12^), and its transcripts levels were significantly positively correlated with platelets (MPV, r = 0.234, *p* = 9.11 × 10^−5^).

### Genetic regulation of liver transcripts and metabolic status

Hepatic genes are involved in a variety of physiological processes. Many transcripts can be regulated through nuclear hormone receptors including retinoid X receptor α (RXRα), liver X receptor (LXR), farnesoid X receptor (FXR), retinoic acid receptor (RAR), constitutive androstane receptor (CAR), pregnane X receptor (PXR) and peroxisome proliferator-activated receptor (PPAR). One peculiar characteristic of some of these receptors is their ability to regulate not only the metabolic system but also the innate and adaptive immune systems^58^,^59^.

We observed a highly significant enrichment of transcripts associated with LXR/RXR, PXR/RXR and FXR/RXR activation among those transcripts correlated with biochemical and body composition traits, consistent with a previous study on liver expression relation to body composition in other pig breeds^6^. These pathways play an important regulatory role in the metabolism of carbohydrates, fatty acids and cholesterol^60^.

Methylmalonic aciduria cblB type (*MMAB*) encodes a protein that catalyzes the conversion of vitamin B12 to adenosylcobalamin. MMAB affects TG levels through adenosylcobalamin and methylmalonyl-CoA mutase^61^. MMAB has been associated with TG levels^62^. In the present study, the transcripts levels of *MMAB* were found to be negatively correlated with TG and *MMAB* exhibited a cis-eQTL.

BUN is the major nitrogenous product of protein and amino acid catabolism and reflects the balance status of amino acids. The protein is often used as an indicator of kidney and liver function. We identified transcripts with cis-eQTL, such as *SORL1, LTBP1*, and *ABHD14B* whose expression was correlated with fat traits and BUN. Low BUN indicates a good balance of amino acids and suggests relatively low urea synthesis and hydration in the liver and a relatively high dietary protein efficiency^63^. Moreover, genetically regulated transcripts such as *SORL1, LTBP1* and *ABHD14B* play a significant role in both metabolic and immune processes. These transcripts might be pleiotropic genes whose genetic pathways are common to obesity, amino acid balance and immune traits.

There is increasing evidence that the complement system plays an important role in diabetes. In particular, mannose-binding lectin (MBL) levels are elevated in diabetes patients^64^. As previously described, *MBL2* transcript levels are significantly positively correlated with platelets, and negatively correlated with creatinine levels. *MASP2* encodes a member of the peptidase S1 family of serine proteases and cleaves complement components C2 and C4 to generate C3 convertase in the lectin pathway of the complement system. Polymorphisms in *MASP2* genes are associated with protein serum levels and functional activity and with susceptibility to or protection against infectious diseases^65^. Significantly higher MASP2 levels are found in children and adults with type 1 diabetes mellitus^66^. In the present study, the expression levels of *MASP2* were found to be positively correlated with leukocytes and negatively correlated with ALB, BUN, TCHO and MFR, further supporting the role of *MASP2* in immune and metabolic processes. Thymosin beta 10 (*TMSB10*) was shown to be correlated with haematological, biochemical and body composition traits, and the transcript levels of *TMSB10* were positively correlated with leukocytes and negatively correlated with erythrocytes, ALB, BUN and all fat traits. This finding is consistent with previous reports of the correlation of *TMSB10* with insulin-stimulated sprouting and adipose tissue expansion^67^.

## Conclusions

In summary, the analysis of transcript profiles together with information on their genetic regulation provide a new resource for understanding genotype-phenotype mapping associated with hepatic gene expression and the physiological processes of immune, metabolic and body composition traits. Analyses of genetically regulated transcripts and the correlations of liver transcript levels with immune and metabolic as well as body composition traits enable the complexity of this system to be captured and visualized. The liver transcripts whose biological and genetically pathways are common to immune and metabolic status were demonstrated. Our analyses of trait-correlated hepatic expression and eQTL detection complement genome-wide association studies for immune and metabolic traits. This detailed analysis highlighted numerous candidate genes common to both systems.

## Material and Methods

### Animals, sample collection and phenotype measure

Animal care and tissue collection procedures followed the guidelines of the German Law of Animal Protection, and the experimental protocol was approved by the Animal Care Committee of the Leibniz Institute for Farm Animal Biology. Performance-tested pigs of German Landrace pigs were used for GWAS of liver transcript levels (n = 297). Liver samples were collected from pigs at an average age of 170 days. Veterinary inspection of the carcasses and organs after slaughter confirmed lack of any impairments, disease symptoms and pathological signs to avoid any bias of blood phenotypes. The haematological and biochemical traits were determined using an automated analyzer device (ABX Pentra 60 HORIBA, Montpellier, France; (Fuji DriChem 4000i, FujiFilm, Minato, Japan).

### SNP genotypes

Genotyping was performed using the PorcineSNP60 BeadChip (Illumina Inc., San Diego, CA, USA) per manufacturer’s SNP Infinium HD assay protocol. In brief, DNA was amplified, fragmented, and hybridized to the PorcineSNP60 BeadChip containing 62163 locus-specific 50-mers. Single-base extension of captured oligos incorporated labels that were detected by Illumina iScan, and images were subsequently converted to intensity data. Intensity data were normalized and assigned a cluster position, genotype, and quality score with GenomeStudio software (Illumina Inc.). Samples with call rates <99% were removed. Markers with low minor-allele frequency (<5%) as well as those that strongly deviated from Hardy-Weinberg equilibrium (p < 0.0001) were also excluded. The average call rate for all samples was 99.8% ± 0.2. The markers of the 60 K chip were mapped to the porcine reference genome, Sscrofa 10.2.

### mRNA microarray analysis

Total RNA was isolated from the liver of 297 animals using TRI Reagent (Sigma, Taufkirchen, Germany). The RNA was amplified using Ambion WT Expression Kit (Affymetrix). Subsequently, 5.5 μg of the resulting cDNA was fragmented and labeled using the Affymetrix Terminal Labeling Kit. The fragmented cDNA was hybridized to the microarray using the Affymetrix Hybridization, Wash and Stain Kit and Affymetrix standard protocols.

Porcine Snowball Microarrays (Affymetrix) containing 47,880 probe-sets were used to determine the expression profile. Expression Console software was used for robust multichip average (RMA) normalization and the detection of present genes by applying the DABG (detection above background) algorithm. Further filtering was done by excluding transcripts with low signals and probes that were present in less than 80% of the samples. 24,909 probes passed the quality filtering and were used for further analyses. Expression data are available in the Gene Expression Omnibus public repository (GEO accession number GSE83932: GSM2221843-GSM2222139).

### Data pre-processing

After quality control and filtering the expression data were further pre-processed to account for systemic effects. Mixed-model analyses of variance using JMP Genomics (SAS Institute) were used for adjustment. The genetic similarity matrix between individuals was computed as identity by descent of each pair for the k-matrix and used as a random effect. For control of population stratification, top principal components (PC) which explain variation of more than 1% were considered as covariates. In total 17 PCs were included as covariates. Gender was considered as a fixed effect, and carcass weight was used as a covariate. The residuals were retained for further analysis.

### eQTL of mRNA

Analyses of eQTLs were conducted using the R-package ‘Matrix eQTL´ for testing the association between each SNP and residual of transcript abundancies by modeling the effect of genotype as least squares model (Shabalin; 2012). ‘Matrix eQTL’ performs a separate test for each gene - SNP pair and corrects for multiple comparisons by calculating FDR^68^. Annotation and localization of SNP sites and probe-sets (Ensembl_Sscrofa_10.2) allowed discrimination of cis- and trans-regulation. We defined an eQTL as ‘cis’ if an associated SNP was located within an area less than 1 Mb from the probe set/gene. The associations of transcript levels with haematological, biochemical and end production traits were evaluated estimating spearman coefficient of correlation (r) and corrected for multiple comparisons by calculating FDR^68^.

### Weighted Gene Co-expression Network Analysis (WGCNA)

A weighted gene co-expression network was constructed using normalized gene expression data of 297 livers with the blockwise Modules function of the WGCNA package in R^69^. The blockwise Modules function allows the entire dataset of 24,909 probe-sets to be utilised in the construction of the weighted gene co-expression network^12^. Module–trait associations were estimated using the correlation between the module eigengene and the phenotype. Within each module, the intramodular connectivity of each gene was evaluated using two methods, defined as module membership (MM) and the soft connectivity (K). Module membership (MM) is defined as the correlation of expression profile (xi) and each module eigengene (ME), 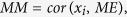 The intramodular soft connectivity (K) is defined as, 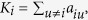 which is the sum of all pairwise adjacencies of a gene to all other genes in the module. Genes within each module were then ranked using both the absolute value of module membership and the intramodular soft connectivity, which enables further identification of key players in the regulation network, defined as hub genes.

## Additional Information

**How to cite this article**: Ponsuksili, S. *et al*. Genetically regulated hepatic transcripts and pathways orchestrate haematological, biochemical and body composition traits. *Sci. Rep.* **6**, 39614; doi: 10.1038/srep39614 (2016).

**Publisher's note:** Springer Nature remains neutral with regard to jurisdictional claims in published maps and institutional affiliations.

## Supplementary Material

Supplementary Table 1

Supplementary Table 2

Supplementary Table 3

Supplementary Table 4

Supplementary Table 5

Supplementary Table 6

## Figures and Tables

**Table 1:** Numerical summary of the whole-genome association study of gene expression levels in liver (eQTL) at significant levels of FDR < 5% (*p* < 10^−7^).

	eQTL (transcripts)
No. of samples	297
Total transcripts	24909
Total SNP	48909
No. of eQTL*	20517 (1401)
No. of *cis*-eQTL**	6865 (1028)
No. of trans-eQTL***	13652 (1040)

^*^Number of genes whose transcript abundance is regulated by the eQTL.

^**^Localization of SNP sites and probe-sets based on Sscrofa_10.2 were considered to define ‘cis´ as windows of 1 Mb.

^***^Number of trans eQTL and eQTL of transcripts which are still not annotated and/or at unknown positions.
