# Supplementary material for: Genetically regulated hepatic transcripts and pathways orchestrate haematological, biochemical and body composition traits
Source: Sci Rep. 2016 Dec 21;6:39614. doi: 10.1038/srep39614 (PMC5175187; doi:10.1038/srep39614)
Supplement: Supplementary Table 1 [file srep39614-s1.doc]

## Genetically regulated hepatic transcripts and pathways orchestrate haematological, biochemical and body composition traits

Siriluck Ponsuksili^1^, Nares Trakooljul^2^, Frieder Hadlich^1^, Fiete Haack^1^, Eduard Murani^2^ and Klaus Wimmers^2*^

1 Research Unit ‘Functional Genome Analysis’, Leibniz Institute for Farm Animal Biology (FBN), Wilhelm-Stahl-Allee 2, D-18196 Dummerstorf, Germany

2 Research Unit ‘Genomics’, Leibniz Institute for Farm Animal Biology (FBN), Wilhelm-Stahl-Allee 2, D-18196 Dummerstorf, Germany

Corresponding author:

Klaus Wimmers

Leibniz Institute for Farm Animal Biology

Wilhelm-Stahl-Allee 2

18196 Dummerstorf, Germany

Phone: +49 38208 68700

Fax: +49 38208 68702

Email: wimmers@fbn-dummertorf.de

Supplementary Table 1.

Definitions of traits, numbers of sample, raw means, standard deviations for each of haematological, biochemical and body composition traits.

| Traits | Definitions of traits | | | | Mean±SD (N=297) | | |
| --- | --- | --- | --- | --- | --- | --- | --- |
| WBC (10³/mm³)  LYM (#)  RBC (106 /mm³)  HGB (g/dl)  HCT (%)  MCV (µm³)  MCH(pg)  MCHC (g/dl)  RDW (%)  PLT (10³/mm³)  MPV (µm³)  PCT(%) | | | White blood cell count  Lymphocytes count  Red blood cell count  Hemoglobin concentration  haematocrit level  Mean Corpuscular Volume  Mean Corpuscular Haemoglobin  Mean Corpuscular Haemoglobin Concentration  Red Distribution Width  Platelets  Mean Platelet Volume  Plateletcrit | 20.06±4.7  8.28±1.9  8.06±0.7  13.75±1.2  43.57±3.2  54.15±2.8  17.11±1.3  31.57±1.4  15.95±1.3  304.32±72.5  7.47±0.5  0.22±0.05 | | |  |
| ALB (g/dl)  NH3 (µg/dl)  BUN (mg/dl)  TCHO (mg/dl)  TG (mg/dl)  GLU (mg/dl)  IP (mg/dl)  CREA (mg/dl) | | Albumin concentration  Ammonia nitrogen concentration  Blood urea nitrogen concentration  Total cholesterol  Triglyceride concentration  Glucose concentration  Inorganic phosphorus concentration  Creatinine concentration | | 4.35±0.3  83.13±19.7  14.31±3.9  85.86±12.7  41.64±13.4  131.50±19.5  8.31±1.2  1.33±0.2 | | |  |
| loin eye area (LEA) [cm^2^] | | area of *M. longissimus dorsi* (Mld) at 13^th^/14^th^ rib | | 43.71±5.9 | |  | |
| fat area (FA) [cm^2^] | | fat area on Mld at 13^th^/14^th^ rib | | 14.08±5.2 | |  | |
| meat to fat ratio (MFR) | | ratio of meat and fat area | | 0.33±0.1 | |  | |
| fat depth at shoulder (FDS) [cm] | | depth of fat and skin on muscle, mean of 3 measures at thickest point | | 3.42±0.5 | |  | |
| fat depth at tenth rib (FDTR) [cm] | | depth of fat and skin on muscle, mean of 3 measures at thinnest point | | 1.70±0.4 | |  | |
| loin fat depth at loin (FDL) [cm] | | depth of fat and skin on muscle, mean of 3 measures at thinnest point | | 1.47±0.4 | |  | |
| average back fat (ABF) [cm] | | mean value of shoulder fat depth, back fat tenth rib and loin fat depth | | 2.20±0.4 | |  | |
| Intramuscular fat content (MLDIMF) % | | Intramuscular fat content of Mld at 13^th^/14^th^ rib | | 1.20±0.5 | |  | |
| Protein content (MLDP) % | | Protein content of Mld at 13^th^/14^th^ rib | | 22.93±0.4 | |  | |
| Body weight (BW) [kg] | | Body weight at slaughter | | 83.95±7.0 | |  | |
| Body length (BL) [cm] | | Body length at slaughter | | 100.54±3.3 | |  | |
|  | |  | |  | |  | |
